# Genome-Wide Screen for *Salmonella* Genes Required for Long-Term Systemic Infection of the Mouse

**DOI:** 10.1371/journal.ppat.0020011

**Published:** 2006-02-24

**Authors:** Trevor D Lawley, Kaman Chan, Lucinda J Thompson, Charles C Kim, Gregory R Govoni, Denise M Monack

**Affiliations:** Department of Microbiology and Immunology, Stanford University, Stanford, California, United States of America; Tufts University, United States of America

## Abstract

A microarray-based negative selection screen was performed to identify Salmonella enterica serovar Typhimurium (serovar Typhimurium) genes that contribute to long-term systemic infection in 129X1/SvJ *(Nramp1^r^)* mice. A high-complexity transposon-mutagenized library was used to infect mice intraperitoneally, and the selective disappearance of mutants was monitored after 7, 14, 21, and 28 d postinfection. One hundred and eighteen genes were identified to contribute to serovar Typhimurium infection of the spleens of mice by 28 d postinfection. The negatively selected mutants represent many known aspects of *Salmonella* physiology and pathogenesis, although the majority of the identified genes are of putative or unknown function. Approximately 30% of the negatively selected genes correspond to horizontally acquired regions such as those within *Salmonella* pathogenicity islands (SPI 1–5), prophages (Gifsy-1 and −2 and remnant), and the pSLT virulence plasmid. In addition, mutations in genes responsible for outer membrane structure and remodeling, such as LPS- and PhoP-regulated and fimbrial genes, were also selected against. Competitive index experiments demonstrated that the secreted SPI2 effectors SseK2 and SseJ as well as the SPI4 locus are attenuated relative to wild-type bacteria during systemic infection. Interestingly, several SPI1-encoded type III secretion system effectors/translocases are required by serovar Typhimurium to establish and, unexpectedly, to persist systemically, challenging the present description of *Salmonella* pathogenesis. Moreover, we observed a progressive selection against serovar Typhimurium mutants based upon the duration of the infection, suggesting that different classes of genes may be required at distinct stages of infection. Overall, these data indicate that *Salmonella* long-term systemic infection in the mouse requires a diverse repertoire of virulence factors. This diversity of genes presumably reflects the fact that bacteria sequentially encounter a variety of host environments and that *Salmonella* has evolved to respond to these selective forces in a way that permits both the bacteria and the host to survive.

## Synopsis

Bacteria belonging to the genus *Salmonella* are capable of establishing a long-term systemic infection in a variety of hosts, including humans, rodents, fowl, and cattle. The ability of *Salmonella* to subvert the active immune response of the host represents millions of years of co-evolution and is the result of specialized virulence factors that promote long-term infection. This study describes a microarray-based genome-wide screen designed to identify genes required by Salmonella enterica serovar Typhimurium (serovar Typhimurium) to persist and replicate in the spleen and liver of mice for up to 28 days. The results demonstrate that serovar Typhimurium utilizes a diverse repertoire of virulence factors, including both known and novel virulence genes, to establish infection and to persist in the host. The authors' data further established a previously unappreciated role for *Salmonella* pathogenicity island 1 in maintaining a persistent systemic infection. In addition, a progressive selection against serovar Typhimurium mutants based upon the duration of the infection was observed, suggesting that certain classes of genes are required at specific times during infection and providing a foundation to further dissect *Salmonella* pathogenesis into distinct temporal phases.

## Introduction

Bacteria belonging to the genus *Salmonella* are capable of infecting a broad range of hosts, including humans, mammals, birds, reptiles, and insects [1]. There are more than 2,400 *Salmonella* serovars that are distinguished serologically based on surface exposed antigens. Serovars belonging to subspecies 1 of Salmonella enterica are responsible for greater than 99% of *Salmonella* infections in warm-blooded hosts [2]. Disease manifestations can range from a self-limiting gastroenteritis to a systemic enteric fever, and have been studied experimentally using animal infection models in mice, cattle, and chickens [3–5].

Another important aspect of *Salmonella* pathogenesis is the establishment of an asymptomatic carrier stage that serves as a reservoir of infection (reviewed in [6]). The carrier stage is characterized by the persistence and replication of the bacteria in the presence of an active adaptive immune response [7–9]. *Salmonella* carriage also provides a prolonged mechanism of transmission due to continued shedding from the host. In a mouse model of persistent infection, viable Salmonella enterica serovar Typhimurium (serovar Typhimurium) are consistently cultured from the spleen, liver, mesenteric lymph nodes, gall bladder, Peyer's patches, and cecum during the first several weeks of systemic infection [8]. After several months, serovar Typhimurium are often still found in these organs and are invariably found within macrophages of the mesenteric lymph nodes [8]. The mechanisms by which *Salmonella* can establish and maintain a persistent infection are complex, since both innate and adaptive immunity must be subverted. This likely reflects millions of years of co-evolution of *Salmonella* with its hosts.

The acquisition of genetic elements (pathogenicity islands, prophage, and plasmids) via horizontal DNA transfer has been central to the evolution of *Salmonella* as a pathogen [10]. The most extensively studied virulence factors are *Salmonella* pathogenicity island (SPI) 1 and 2 (SPI1 and SPI2). Each SPI encodes a membrane-associated type III secretion system (T3SS), which promotes the translocation of several virulence effector proteins believed to contribute to bacterial entry into and replication within host cells [11]. The contribution of SPI1 to *Salmonella* pathogenesis has been proposed to be limited to the gastrointestinal phase of disease in a naive host in which it facilitates *Salmonella* breaching the epithelial surface of the Peyer's patches to encounter the host innate immune system, including macrophages and dendritic cells [11]. The initial interactions between *Salmonella* and macrophages generally result in caspase-1–dependent host cell death mediated by one or more SPI1-secreted effector proteins [12], which may aid in the establishment of systemic disease [13].

In order for the infection to extend beyond the intestinal mucosa, *Salmonella* must replicate and persist within macrophages, a privileged niche which allows *Salmonella* to elude the adaptive immune response [14–16]. *Salmonella* virulence genes encoded by SPI2 have evolved to allow intracellular bacteria to subvert the bacteriocidal properties of macrophages and to create a specialized *Salmonella*-containing vacuole in which it can replicate [17]. Experimental evidence from several laboratories suggests that *Salmonella* resides within macrophages most of the time during systemic infection [8,18,19].

Much of what is known about *Salmonella* virulence factors has been determined using an experimental infection model based on the *Nramp1* susceptible *(Nramp1^s^)* mouse strains BALB/c and C57Bl/6 (Nramp1 is nonfunctional). Nramp1, also called Slc11a1, is a macrophage-specific protein that localizes to the membrane of the *Salmonella*-containing vacuole and removes cations from the vacuole, which aids in the control of *Salmonella* replication [20]. Macrophages from *Nramp1^s^* mice allow for an increased rate of serovar Typhimurium replication in vitro compared to *Nramp1^r^* (Nramp1 is functional) macrophages [21]. The inability of *Nramp1^s^* mice to control wild-type *Salmonella* replication likely explains why these mice typically succumb to an overwhelming systemic infection 7–10 d postchallenge [20,22]. Our laboratory has recently described a *Salmonella* persistence model based on the mouse strain 129X1/SvJ *(Nramp1^r^),* in which mice infected with serovar Typhimurium typically do not succumb to infection and bacteria can be recovered from systemic sites up to one year after infection [8]. This persistence model closely resembles the carrier state seen in human typhoid fever and provides the opportunity to better study the basic aspects of persistent bacterial infections.

The contribution of serovar Typhimurium genes has been largely unexplored in the mouse model of persistent infection. The known *Salmonella* persistence proteins are Mig-14, VirK, and AceA. Mig-14 and VirK are important for resistance to host antimicrobial peptides [23,24], whereas AceA is a key enzyme in the glyoxylate pathway [25]. The goal of the current study was to identify novel genes that allow *Salmonella* to establish and maintain long-term systemic infection. To this end, we used a microarray-based genetic screen to identify genes used by serovar Typhimurium to infect the spleens of mice for up to 28 d [26,27]. The results indicate that serovar Typhimurium utilizes a diverse array of virulence factors to colonize systemic sites and possesses classes of genes for distinct stages of infection. Furthermore, we demonstrate that the SPI1-encoded T3SS and the invasion effectors/translocases SipB, SipC, and SipD, as well as the SPI2 T3SS-secreted effector SseK2 and SPI4, have a previously unappreciated role in long-term systemic disease.

## Results/Discussion

### Microarray-Based Negative Selection Strategy Reproducibly Identifies Genes Required for the Establishment of a Systemic Infection

**Figure 1:**
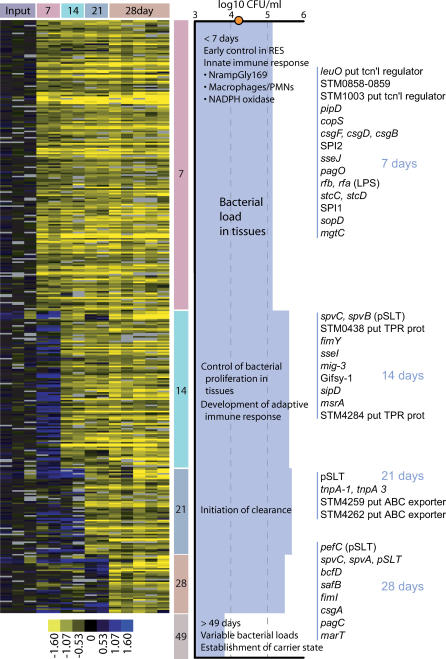
Time-Dependent Negative Selection of Serovar Typhimurium Mutants SAM two-class analysis was performed grouping various time points in order to identify the features that are most significantly different between time points. The data corresponding to these SGLs were manually assembled and verified to have the expected hybridization patterns, and then duplicate features were removed. The arrays were organized by time point and the features placed in genome order. Colored bars at the top indicate grouping of technical microarray replicates for the same time point, and the same color scheme was employed to delineate groups of features that exhibit the same time-dependent pattern (vertical bars on right of microarray data figure). Also indicated is the average splenic log10 CFU/ml bacterial load observed over the course of infection along with the predicted stages of the host immune response (S. Clare and G. Dougan, unpublished data). The orange circle on the *y*-axis indicates the initial inoculum of 1.6 × 10^4^ CFUs. Bacterial loads were comparable between liver and spleen. The microarray data in this figure have been made available in a format that can be viewed in both Treeview and Excel (Dataset S1).

In this study, we wished to identify novel genes that are important for establishment and maintenance of a 28-d systemic infection in mice. This infection model, for reasons discussed below, required the mice to be inoculated with approximately 5 × 10^4^ bacteria intraperitoneally (IP). In this model, bacterial numbers increase modestly for the first 21 d and remain about the same through 28 d (Figure 1). During this acute phase of infection, the histopathological lesions were frequent in the spleen and liver, and contained large numbers of polymorphonuclear lymphocyte (PMN) cells, monocytes/macrophages, and regions of necrosis (data not shown). Only between 28 and 49 d were the bacterial numbers significantly reduced (Figure 1), after which bacterial persistence is established. The liver and spleen tissues from mice infected for 49 d contained typical microgranulomas, which contained PMNs and central regions of necrosis. We used this model of infection to identify novel serovar Typhimurium virulence genes that are required to infect mouse spleens for 28 d. The contribution of these genes to persistence in the reticuloendothelial system beyond 28 d will require additional studies.

A Tn10 transposon library consisting of 50,000 individual mutants [26] was inoculated IP into 129X1/SvJ mice to identify serovar Typhimurium genes that contribute to the establishment and maintenance of a long-term infection in mice. Each transposon insertion contains a T7 transcriptional promoter. When purified bacterial genomic DNA isolated from a mixed bacterial population containing specific transposon inserts is subjected to an in vitro transcription reaction, RNA is generated corresponding to each transposon insertion [27,28]. Of course, a transposon insertion may disrupt transcription of downstream genes, resulting in polar mutations. In any event, mutants initially present in laboratory culture that cannot survive the infection process disappear from the bacterial pool isolated from an infected animal and will no longer have RNA generated from their T7 promoters. In this manner, probes corresponding to each transposon insertion is generated, and by comparing the input library with the output obtained from mice, genes potentially important for the establishment and maintenance of infection can be identified. Specifically, we inoculated the laboratory-grown library into two groups of mice. In the first group (passage 1), 50 mice were injected with 1.6 × 10^4^ colony-forming units (CFUs) and at day 7 (ten mice), 14 (ten mice), 21 (ten mice), 28 (eight mice), and 49 (four mice) postinfection mice were sacrificed and bacteria were recovered from their livers and spleens. In the second group (passage 2), ten mice were injected with 7.9 × 10^3^ CFU. The seven surviving mice were sacrificed at 29 d postinfection, and bacteria were recovered from their livers and spleens. The spleen outputs for each time point from passage 1 were pooled and processed for microarray analysis whereas both pooled and individual samples from both liver and spleen were processed from passage 2.

**Figure 2:**
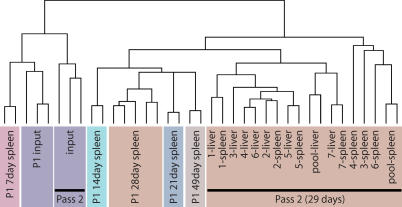
Microarray Dendogram Illustrating the Relationship between the Different Time Points and Passages The dendogram was generated from a single dataset which included arrays from both mouse passages. For the first passage, spleen pooled arrays from 7, 14, 21, 28, and 49 d postinfection, as well as input arrays, were included and are indicated by the prefix “P1.” For the second 29-d passage, spleen and liver pooled arrays, input arrays, and individual mouse tissue arrays were included. Arrays corresponding to the same time point are grouped according to color boxes.

Any screen utilizing a pool of mutants is subject to a number of variables that can dramatically affect its reproducibility [29]. The choice of infection route is critical as physical and biological bottlenecks can greatly diminish the complexity of the library in a stochastic manner. For our experiments, we inoculated mice IP because previous studies have shown that the oral route imposes a significant bottleneck in which less than 1% of bacteria were able to successfully breach the intestinal barrier and colonize systemic tissues [30–32]. Although it is possible that subsequent to an IP inoculation, a bottleneck exists that can affect the number of bacteria that successfully disseminate from the peritoneal cavity, the IP route has been shown to have minimal impact on the reproducibility of a library screen [17,29]. In order to address any concerns regarding the possible impact that the IP route of infection may have on our ability to identify virulence genes, we analyzed the data from the two independent passages utilizing the Statistical Analysis of Microarrays software (SAM) tool [33], a program that analyzes sets of microarrays in order to identify the genes that differ in the most statistically consistent manner. Application of SAM to a dataset comparing the individual liver and spleen samples from the 29 d second passage revealed that there were no differences between the two tissues (Figure S1). Furthermore, when all the arrays were clustered, we observed that the liver and spleen samples did not segregate into distinct nodes and that the liver and spleen samples from the same mouse often shared the same node (Figure 2). This indicates that there is not a bottleneck that differentially affects the dissemination from the peritoneal cavity to the spleen compared to the liver. Finally, we have always observed a strong correlation in the bacterial loads in spleens and liver, suggesting that there are not significant differences in bacterial replication between the two tissues. Taken together, these findings indicate that the impact of the IP route of inoculation on the complexity of the library is negligible under our experimental conditions. Furthermore, these data indicate that there are not any specific virulence factors for replication/survival in the liver versus the spleen.

**Figure 3:**
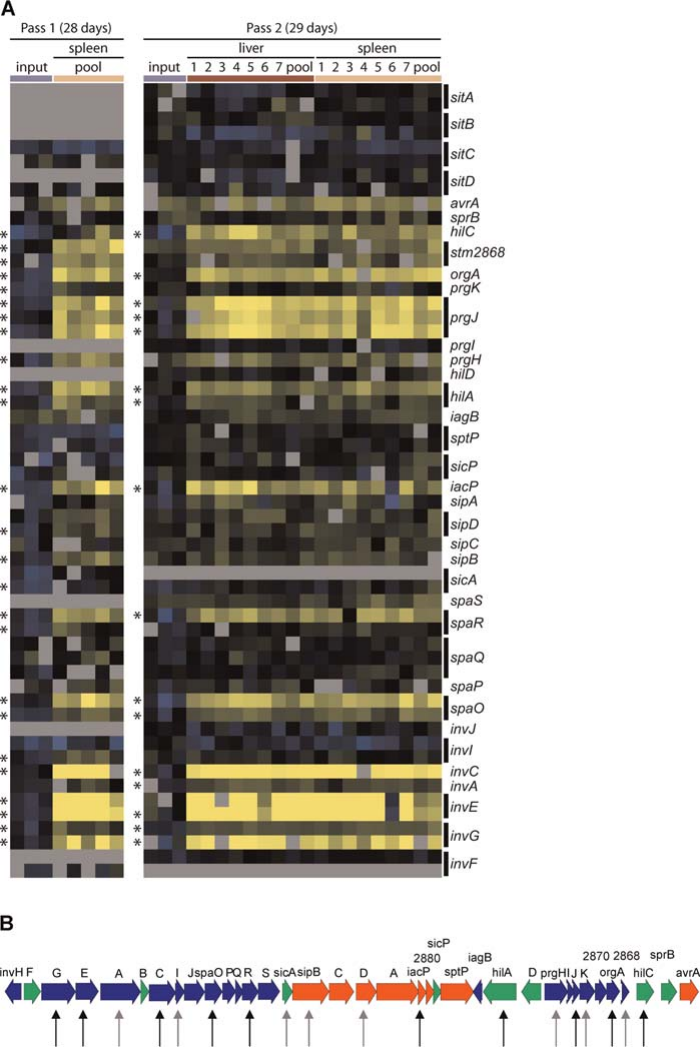
SPI1 Genes That Were Negatively Selected (A) The features corresponding to genes in the SPI1 were extracted from each of the passage 1 and passage 2 datasets. Each row represents a feature presented in genome order. Each column corresponds to an array, and columns are grouped by color bars to indicate either technical replicates or samples from the same type of organ. Passage 1 arrays include spleen pooled arrays and input arrays whereas passage 2 arrays include input, spleen, and liver pooled, and individual mouse tissue arrays (indicated by numbers) and are labeled at the top of each panel. Asterisks along the left of each panel correspond to features that were identified by SAM analysis as being significant. Black bars along the right group together the features that correspond to the same gene. Grey indicates features that have missing data, blue or black indicate mutants that are still present in the sample relative to the library reference, and yellow indicates mutants that are absent in the sample relative to the library reference. (B) Genetic map of SPI1 illustrating genes identified as contributing to systemic disease for 28 d postchallenge. Vertical arrows below ORF indicate specific genes identified in the screen, with black arrows showing those identified in both passages and grey arrows showing those genes identified in one passage. ORF are colored to represent function: blue represents T3SS; green, regulartory/chaperone; and red, secreted effectors.

The initial complexity of the library is also important and can affect the ability to reproducibly detect mutants [17,29]. We assessed the amount of reproducibility between the two biological replicates to determine if the parameters of our experiments were at sufficient levels for the identification of mutants attenuated in the ability to establish or maintain a long-term systemic infection. The top 300 negatively significant features (representing mutants that are absent from the mouse-selected pools) in both passages were identified by performing a SAM analysis comparing the input and spleen pooled arrays from the first passage, and the input and all samples from the second passage. This analysis did not identify any positively significant features. There are a total of 138 overlapping features corresponding to 46% of each of the original list of 300. These features represent a total of 118 genes (as there are multiple features for many of the genes on the array) and two intergenic regions (Table S1). In addition, although the exact same feature may not have appeared in the overlapping dataset, oftentimes different features corresponding to the same gene or corresponding to the same known or putative transcriptional unit appeared in the compiled list from either passage. The negatively selected genes belonging to the same known or putative transcriptional unit have been highlighted in Table S1. Such information is valuable because gene products encoded within the same operon generally function in the same biological process. Therefore operons containing multiple negatively selected genes may contain additional genes that are important during systemic disease but were not identified in the screen. Obvious examples include the LPS (i.e., *rfb* and *rfa*), fimbrial, and T3SS operons. As an illustration, we extracted all the data for SPI1 from both datasets and compared the hybridization patterns between the two biological replicates (Figure 3). The hybridization patterns are highly reproducible, giving us further confidence in the negative selection technique.

Given the high complexity of our library as well as the dynamic and complicated nature of the mouse as a selection system, the result of a 46% overlap between the two independent passages indicated to us that the negative selection strategy and conditions that we used are sufficiently robust for the identification of novel genes important for the establishment and maintenance of infection.

### Functional Classification of Negatively Selected Genes

The 118 genes and two intergenic regions corresponding to the 138 features identified as negatively selected in both passages represent a wide diversity of genes. These genes are referred to as the significant gene list (SGL). Sixty-four of them (53%) encode for proteins with putative or unknown functions as defined in serovar Typhimurium strain LT2 genome annotation [34] suggesting that these may represent uncharacterized genes with novel functions in the establishment and maintenance of infection during the first 28 d postchallenge. The insertions in pseudogenes could affect regulatory elements (e.g., plasmid-encoded PSLT071 likely affects plasmid stability) or have polar effects on adjacent genes. In addition, many of these genes may represent mutants that are compromised for the acquisition, utilization, or production of key nutrients but are not selected against until they are in the host (see column I in Table S1). Finally, it should be noted that mutants defective for survival in the peritoneal cavity or for dissemination from the peritoneum to the spleen (e.g., serum-sensitive mutants) would also be scored as negatively selected in this screen.

To assess the functional groups to which the genes belong, the overlapping negative significant genes were associated with their Clusters of Orthologous Groups of proteins (COG) designations available for the serovar Typhimurium LT2 genome [34]. Each gene product was therefore assigned to a functional class based on whether it possesses a homolog within a large database representing all domains of life (Table 1) [35]. Overall, this comparison demonstrated that the mutants negatively selected in mice represent many facets of *Salmonella* physiology (Table 1). This result is similar to the findings from a screen for the genetic requirements for Mycobacterium tuberculosis persistence [36]. In our dataset, the majority of functional classes and the proportion from each class did not change significantly between the negatively selected genes and the initial input dataset, indicating that no specific functional classes were selected against during infection (Table 1).

**Table 1:**
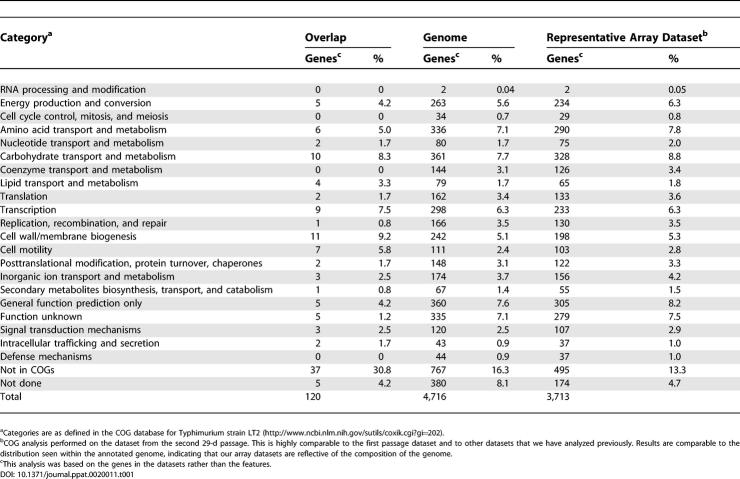
COG Breakdown for Serovar Typhimurium Negative Significant Genes

The major difference between the negatively selected genes and both the complete dataset and genome distribution is the increase in the size of the “not in COGs” category (31% in the overlap list compared to 16% in the genome). This category corresponds to gene products that are hypothetical or are not widely conserved and therefore have no functional counterparts in the database. These could be gene products whose functions have never been characterized, or they could also correspond to genes that are unique to *Salmonella* or serovar Typhimurium [35]. This observation, in combination with the fact that many of the genes identified are of unknown or putative function, suggests that there is much that needs to be characterized with regard to serovar Typhimurium's genetic requirements during the establishment and maintenance of a long-term infection. Notably, the genes in the negative SGL that fall into the “not in COGs” category are significantly enriched for genes of putative function in addition to genes corresponding to DNA that was horizontally transferred to the *Salmonella* genome via mobile DNA, such as the prophages, the virulence plasmid, and pathogenicity islands (Table S1). These genetic elements therefore require a more direct analysis to determine their contribution to systemic disease.

### A Diverse Repertoire of Virulence Genes Contributes to Long-Term Systemic Infection

#### Pathogenicity islands.

When the genes from the negative SGL were arranged in genome order, we observed that mutations in 13 SPI2 genes were selected against in both passage 1 and passage 2, consistent with the known role of this genomic region in intracellular replication and survival of serovar Typhimurium within the host [14,15] (Table 2 and Table S1). Lexical analysis of the microarray dataset revealed that the SPI2 genes were unlikely to be over-represented in the SGL by chance (*p*(*X* ≥ 19) ≪ 10^−10^). The regulatory gene *ssrA* and genes encoding structural components of the SPI2 T3SS were represented. Mutations in the genes that encode the SPI2-secreted effectors SseK2, SseJ, SseI, SsaE, SseB, and SopD2 were also selected against. A role in systemic disease has been demonstrated for SopD2, SseJ, SsaE, and SseB [15,37,38]; however, this is the first time SseI and SseK2 have been implicated in contributing to systemic disease in mice [38,39].

**Table 2:**
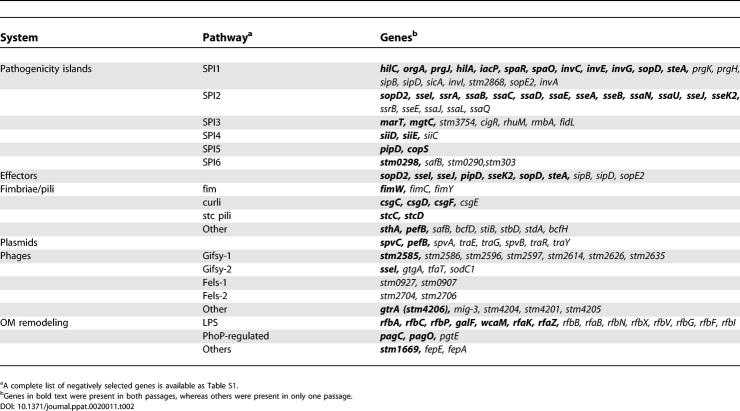
Summary of Negatively Selected Serovar Typhimurium Genes

Twelve genes associated with SPI1 were present in the negative SGL, including genes that encode regulatory proteins and components of the T3SS needle structure, as well as the secreted invasion effectors SipB and SipD (Table 3 and Figure 3). The presence of 12 genes in the SGL is highly significant (*p*(*X* ≥ 18) < 5E10^−9^). In addition, bacteria with mutations in *sopD* and *steA,* genes that encode SPI1-secreted effectors, were negatively selected against. SopD and SteA have recently been shown to be important for *Salmonella* replication in the spleen of *Nramp1^s^* mice [40,41], whereas this is the first implication of a role for the SPI1-encoded T3SS, SipB, and SipD during systemic infection.

**Table 3:**
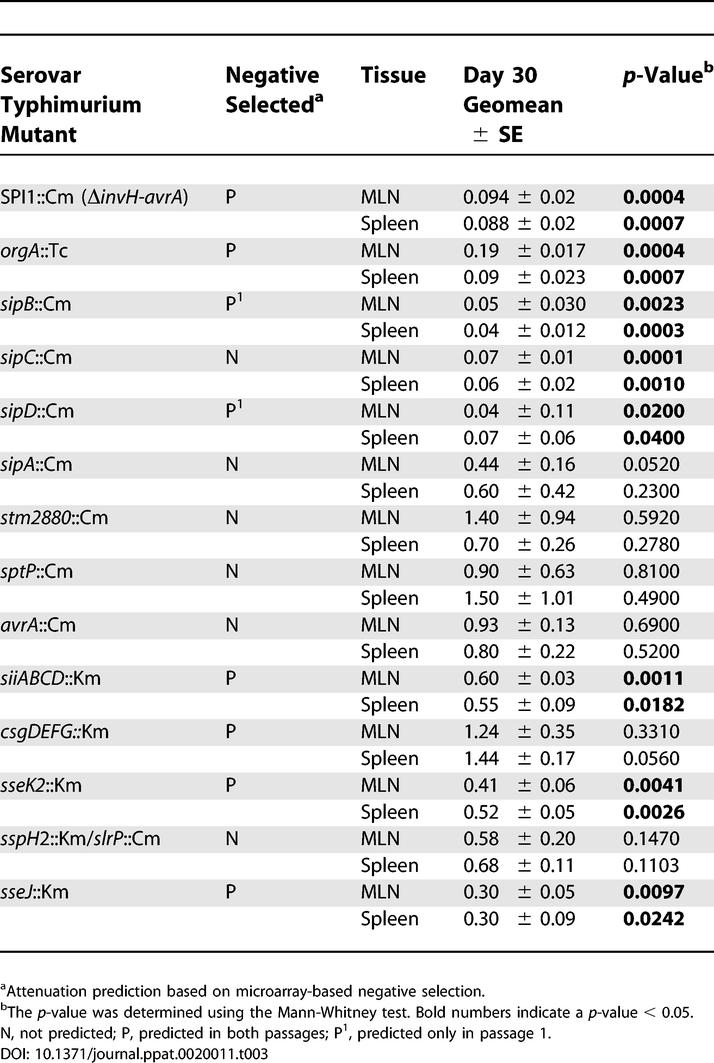
Summary of Data from CI Experiments That Tested Serovar Typhimurium Mutants for Long-Term Colonization in the Spleens and MLN of 129X1/SvJ Mice

SPI3 is an approximately 17-kb genetic locus that encodes a magnesium uptake system that is important for intracellular survival and systemic disease in mice [42]. The magnesium system, *mgtC,* was identified in this screen as well as mutations in *marT*. The *marT* gene encodes a protein homologous to the transcriptional regulatory protein ToxR from Vibrio cholerae [43]. Several additional genes within SPI3 were selected against during only one passage and may prove to be important for systemic disease (Table 2).

The SPI4 operon was recently identified in a signature-tagged mutagenesis screen as required for disease in calves but not chicks [44]. Our results corroborate a role for this operon in disease as mutations in *siiC* (STM4259), *siiD* (STM4260), and *siiE* (STM4261) were negatively selected (Table 2). The genetic organization of these genes is typical of type I secretion systems, such as alpha hemolysin of Escherichia coli. The *siiC* gene encodes an outer membrane (OM) protein homologous to TolC. The *siiD* gene encodes a protein that is homologous to members of the HlyD protein family. The *siiE* gene encodes a large protein (5,559 amino acids) with a low level of similarity to RTX toxins [44].

Both SPI5 and SPI6 were previously shown to be dispensable for systemic infection in *Nramp1^s^* mice [45,46]. However, in our screen, the SPI5 genes *pipD,* which encodes a cysteine protease homolog, and *copS,* which encodes a histidine kinase, contributed to long-term systemic infection. The SPI6-encoded fimbrial chaperone *safB,* as well as three other genes within SPI6 that encode putative proteins, were negatively selected. These observations suggest that the persistence model may be better suited to study these particular pathogenicity islands during systemic disease.

#### Fimbriae/pili.

Fimbriae are filamentous, extracellular appendages that are ubiquitous among the Enterobacteriaceae*.* Fimbriae are generally associated with bacterial adherence to cells or surfaces, facilitating either bacteria–host or bacteria–bacteria interactions [47]. There are 13 fimbrial operons within the serovar Typhimurium LT2 genome [34], however, the specific functions of these fimbriae during *Salmonella* pathogenesis are not known. The negative SGL contained eight fimbrial genes that were identified in both passages and represent five types of fimbriae: *csg, pef, fim, sth,* and *stc* fimbriae. During the first passage, mutations that would compromise the *saf* and *bcf* pili were selected against, and during the second passage mutations that would compromise the *sti, std,* and *bcf* pili were selected against.

The role of *Salmonella* fimbriae during intestinal colonization has been studied mainly in *Nramp1^s^* mice, in which they are important for adhesion to the small intestine and Peyer's patches [48]. Recently, the *lpf, bcf, stb, stc, std,* and *sth* fimbrial operons were reported to contribute to intestinal persistence of serovar Typhimurium in *Nramp1^r^* mice [49]. In our study, mutations in the *stc (stcC* and *stcD), bcf (bcfD),* and *sth (sthA)* operons were selected against, indicating that these particular fimbriae contribute to both gastrointestinal and long-term systemic infection. Thus, *Salmonella* possesses a wide array of fimbriae that contribute to various stages of colonization and persistence; however the exact function of these fimbriae during a long-term systemic infection will require additional studies.

Several genes previously associated with systemic disease and persistence were not identified in this screen. Most notable are the *mig-14* (not present on microarray) [23], *aceA* [25], and *sifA* [50] genes. One possible explanation is that the deficiency of these mutants may be complemented by the wild-type gene product within a complex mutant population. Therefore, the absence of known virulence genes from the negative SGL would imply that although the list of virulence genes is long and instructive, it is likely not complete.

Although several virulence factors described here have already been demonstrated to be important for *Salmonella* virulence in mice, many of the genes identified in this study have not been previously implicated in systemic disease. Furthermore, previous genetic screens for *Salmonella* virulence genes have not identified a comparable or as comprehensive negative selection of bacterial genes [17,26]. For example, using the same methodology as described in this study but using BALB/c mice *(Nramp1^s^),* the only virulence genes in common are those in the SPI2 and LPS operons [26]. One explanation for the discrepancies between the genetic requirements for *Salmonella* is the genetic differences between the mouse strains, with the most likely candidate being the *Nramp1* locus [20].

*Nramp1*-sensitive macrophages allow for an increased rate of *Salmonella* replication in vitro compared to *Nramp1*-resistant macrophages [21]. The inability of *Nramp1^s^* mice to control wild-type *Salmonella* replication likely explains why these mice typically succumb to infection 7–10 d postinfection [20,22,51]. In addition, the *Nramp1* locus is responsible for controlling *Salmonella* virulence gene expression [52]. Therefore, the status of the host *Nramp1* locus affects the length of infection and *Salmonella* gene expression, both of which could account for the different *Salmonella* genetic requirements for infection in *Nramp1^r^* and *Nramp1^s^* mice. The differences between these models do not necessarily indicate that the current description of *Salmonella* pathogenesis is incorrect but rather emphasizes that many aspects have yet to be investigated.

### Selection against Serovar Typhimurium Mutants Correlates with the Length of Infection

In order to examine the overall relationship between serovar Typhimurium mutant populations from the different timepoints, hybridizations representing pooled spleen samples from days 7, 14, 21, 28, and 49 postinfection were subjected to a hierarchical cluster analysis (Figure 2). In general, all technical replicates from a given time point share a distinct node, which indicates that they behave similarly yet are distinct from other time points. Analysis of the 49-d samples suggests that little negative selection occurred between 28 and 49 d. Furthermore, the bacterial loads from spleens and livers at day 49 were very low in comparison to the levels at the other time points (Figure 1). The decreased complexity of the library at day 49 due to the low bacterial burden might promote the false detection of negatively selected genes and prompted us to remove this time point from further analysis. The time-dependent difference in the *Salmonella* mutant populations (Figure 2) suggests that there was a progressive gene loss in these populations that may reflect specific classes of virulence genes (Figure 1 and Table 2). During the first week of infection, 165 genes were negatively selected. The day 7 list of negatively selected genes includes most of the components of SPI1 and SPI2, a few of their respective secreted effectors such as *sopD* and *sopD2,* and LPS biosynthesis genes (Table 2). At 28 d, it is notable that four different pili/fimbriae, as well as the OM proteins *pagC* and *bigA,* were selected against at this time. Although the significance of this is unknown, it may suggest that OM/surface alterations are particularly important at this time point.

The concept that classes of virulence genes are important for different stages of infection was proposed for M. tuberculosis during experimental mouse infections [36,53]. Our data indicate that there are also classes of *Salmonella* virulence genes important for different stages of persistence. Although it is intriguing to speculate that as the host develops an immune response to *Salmonella,* the bacteria possess a specific repertoire of genes for the particular stages of infection, we do not have any specific data that suggest that this is the case. More comprehensive analysis of individual serovar Typhimurium mutants should be performed to accurately classify virulence genes, which will serve to further refine our understanding of the mechanisms that *Salmonella* employs to persist within animal hosts.

### SPI1-Secreted Invasion Effectors, SPI2-Secreted Effectors, and SPI4 Contribute to Long-Term Systemic Infection

We sought to further investigate the role of individual virulence factors identified in the microarray screen that have not previously been implicated in systemic disease. To address this, competitive index (CI) experiments were performed in which the relative fitness of an individual serovar Typhimurium mutant could be compared to that of wild-type bacteria within a single mouse. For each CI experiment, an equal ratio of wild-type serovar Typhimurium and each mutant was introduced into 129X1/SvJ mice by IP injection. Although this is not the normal route of *Salmonella* infection, we find, using the carriage model, comparable bacterial loads in the liver and spleen after 30 d of infection regardless of the mode of infection, oral or IP (data not shown).

The CI values are based on bacteria isolated from the mesenteric lymph nodes (MLN) and spleen at 30 d post-infection. The results demonstrate that mutants in two SPI2-secreted effectors, *sseJ*::Km and *sseK*2::Km, and a mutant that is deleted for the majority of SPI4, Δ*siiABCD*::Km, were out-competed by wild-type bacteria by 30 d postinfection in both the spleen and MLN (Table 3). A requirement for these virulence factors as determined with the CI is therefore consistent with that predicted in the microarray-based screen (Table 2).

The microarray screen predicted that the curli pili proteins CsgDEFG contribute to long-term systemic infection; however, the *csgDEFG* mutant did not display a significant disadvantage in the CI experiments (Table 3). This observation is not consistent with the microarray screen and raises the possibility that there may be false positives in the gene list, a concern associated with any global screen [29]. However, it is intriguing that genes from the curli operon were identified independently in biological replicates, raising the possibility that the differences in the infection conditions (CI experiments versus selection experiments) may affect the selection pressures. It must therefore also be considered that although the infection experiments are similar in many respects, one major difference between them is that the representation of each mutant is significantly altered (50% of population in CI versus about 0.5% in negative selection) and may therefore impact the negative selection pressures. This idea would stress the need for further investigation of the various virulence genes in vivo.

In order to further dissect the role of SPI1 during systemic disease, ten serovar Typhimurium SPI1 mutants were chosen that are either deficient in the translocation of all effector proteins or individual effector proteins: SPI1::Cm (Δ*invH-avrA,* removes entire SPI1), *orgA*::Tet (T3SS needle component) [54], *sipB*::Cm (invasion effector), *sipC*::Cm (invasion effector), and *sipD*::Cm (invasion effector), all of which are deficient for the translocation of all SPI1 effector molecules, and the individual SPI1 effector mutants *sipA*::Cm (actin binding/neutrophil attraction), *stm2880*::Cm (unknown function), *sptP*::Cm (GTPase-activating protein/phosphatase), *avrA*::Cm (cyteine protease), and *slrP*::Cm/*sspH2*::Km (Leucine-rich repeat–containing proteins) (because SlrP and SspH2 share 40% identity, a double mutant was used to avoid any functional redundancy that could interfere with the analysis) (for a review of SPI1 biology see [11]).

**Figure 4:**
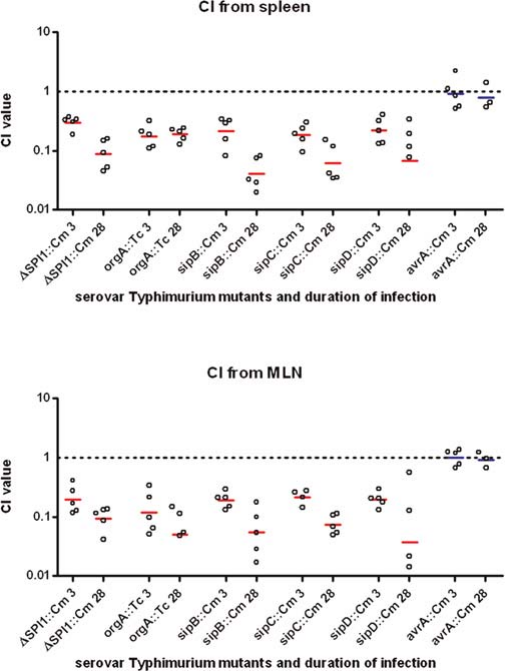
CI Time-Course Demonstrating the Requirement of the SPI1 Effectors SipB, SipC, and SipD for the Establishment and Maintenance of Infection in the MLN and Spleens of *Nramp1^r^* Mice Horizontal bars indicate the geometric means of CI values, and individual CI values are illustrated with open circles. The geometric means and standard errors for each mutant at day 3 in the MLN and spleen are: ΔSPI1, 0.20 ± 0.06 and 0.30 ± 0.03; *orgA*::Tc, 0.12 ± 0.06 and 0.17 ± 0.04; *sipB*::Cm, 0.19 ± 0.03 and 0.21 ± 0.05; *sipC*::Cm, 0.22 ± 0.03 and 0.18 ± 0.04; *sipD*::Cm, 0.20 ± 0.03 and 0.22 ± 0.05; *sseJ*::Kan, 0.32 ± 0.50 and 0.24 ± 0.07; and *avrA*::Cm, 1.01 ± 0.14 and 0.91 ± 0.32. Those values for day 30 are given in Table 3. Geometric means in red indicate attenuated mutants whereas those in blue are not as assessed by the Mann-Whitney test.

The CI values are based on bacteria isolated from the MLN and spleen at 30 d postinfection (Figure 4). The results demonstrate that the SPI1::Cm, *orgA::*Tc, *sipB*::Cm, *sipC*::Cm, and *sipD*::Cm mutants were out-competed by wild-type bacteria by 30 d postinfection in both the spleen and MLN (*p*-value < 0.05 using the Mann-Whitney test). A requirement for these virulence factors as determined with the CI is therefore consistent with that predicted in the microarray-based screen, except for *sipC*::Cm, which was not predicted (Table 2). We were therefore able to predict that sipC contributes to systemic disease, based on the location of the *sipC* gene between the *sipB* and *sipD* genes on the same transcript.

In addition, the CI values for the ΔSPI1::Cm, *orgA*::Tc, *sipB*::Cm, *sipC*::Cm, and *sipD*::Cm mutants display comparable kinetics (Figure 4) in which the CI values drop over time (except *orgA* in MLN). The consistent drop in the CI may suggest a continuous requirement for these virulence genes over the course of systemic infection. These kinetics are distinct from those of the *sseJ*::Km mutant experiment, which gave similar CI values at day 3 (0.32) and day 30 (0.24) postinfection (Table 3 and Figure 4).

In contrast, the *sipA*::Cm, *stm2880*::Cm, *sptP*::Cm, *avrA*::Cm, and *slrP*::Cm/*sspH2*::Km mutants do not display a competitive disadvantage compared to wild-type bacteria for up to 30 d (Table 3), indicating that these gene products do not have an obvious role during long-term systemic infection. The observations with the *sipA, stm2880, sptP, avrA, slrP,* and *sspH2* mutants are consistent with the microarray screen, in which these genes were not predicted to be important for long-term systemic infection. Importantly, these results show that the presence of the chloramphenicol resistance cassette had no effect on serovar Typhimurium virulence.

One of the major findings of this work is the demonstration that SPI1 is important for systemic infection, which challenges the prevailing model that SPI1 only contributes to the gastrointestinal phase of *Salmonella* pathogenesis [11]. Indeed, others have demonstrated in streptomycin-pretreated mice that the role of SPI1 in the localization of serovar Typhimurium to Peyer's patches may not be required to establish systemic disease in mice and that SPI1 may have alternative functions during infection [55,56]. Likewise, SPI2 has been proposed to be expressed in the lumen of the intestine and has been implicated in intestinal inflammation, suggesting that the functions of virulence factors during *Salmonella* pathogenesis may not be as segregated as once believed [57]. The results presented here demonstrate that the SPI1-encoded T3SS and invasion complex, comprised of SipB, C, and D, play an important role in establishing and maintaining the systemic state of infection. We can rule out the SPI1-secreted effectors SipA, SptP, AvrA, and SlrP as being responsible for these functions. However, several SPI1-secreted effectors remain to be tested before we can conclude which virulence proteins are responsible for these functions.

It has been proposed that SPI1-encoded products are not required for intracellular survival but are required for entry of *Salmonella* into host cells [58]. Although this would suggest distinct roles for SPI1 and SPI2 during pathogenesis, there is mounting evidence that the contributions of SPI1 and SPI2 to the multiple stages of pathogenesis may not be mutually exclusive. For example, it has been observed that SPI2 mutants have altered SPI1 gene expression and invasion properties [59,60], perhaps mutations in SPI1 have similar pleiotrophic effects. Still others have shown that SPI1 may mediate important events early after invasion that help establish a permissive intracellular niche [61,62]. Most recently, it has been shown that some SPI1-secreted effectors, including SptP and SopB, persist within host cells after invasion, suggesting that there is continued secretion of these effectors postinvasion [63,64]. Consistent with these observations, we have observed an enrichment of SPI1 mutants in a negative SGL derived from serial passage of the transposon-mutagenized library in RAW 264.7 macrophage cells [26]. Therefore, there appears to be considerable functional overlap between SPI1 and SPI2 during pathogenesis.

The precise role(s) of SPI1 during long-term systemic *Salmonella* infection and persistence are not known. Perhaps SPI1 is required to maintain colonization of the Peyer's patches, which results in continual seeding of the systemic sites, such as the MLN, spleen, and liver, during long-term infection. Indeed, we observe that serovar Typhimurium can colonize the Peyer's patches of mice infected by the IP route (unpublished data). Alternatively, the SPI1 deficiency observed during persistent infection is related to *Salmonella*'s inability to reinvade new host cells or to reestablish a replicative state within host cells. Host cell death is a hallmark of *Salmonella* infection of the liver and spleen [65] and may result in bacteria being released into the extracellular environment. In order to maintain its privileged niche, *Salmonella* would need to effectively reinvade or reestablish itself within a host phagocyte. SPI1 also mediates caspase-1–dependent macrophage cytotoxicity that results in the release of proinflammatory cytokines that lead to the recruitment of immune cells [13,66], which may serve as new cells for *Salmonella* to infect. Alternatively, the role of SPI1 may be even more complex, involving modulation of host responses or integration with bacterial signaling pathways from within the host cell. Regardless, the SPI1 deficiency is mirrored in the spleen and MLN (Figure 4), indicating a role for SPI1 in maintaining *Salmonella* infection of systemic sites.

It is emerging that the majority of in vivo infected phagocytes contain low bacterial numbers of *Salmonella* (1–3/phagocyte) and that most, but not all, bacteria are conclusively intracellular [8,18,19]. It has been proposed that *Salmonella* spread at systemic sites by increasing the number of infection foci rather than expansion of initial foci [19,67]. Although the exact mechanism by which the bacteria spread to new foci is not known, it is possible that *Salmonella* exists for some period of time as an extracellular form, either in the intestine or in systemic tissues, during persistence. SPI1 may play a role in mediating the increase in the number of infection foci. In addition, genes from SPI4 and SPI5, which are down-regulated during replication in macrophages in vitro [68], as well as a number of fimbrial genes, appear to contribute to persistence (Table 2). Taken together, these observations imply that *Salmonella* persistence relies upon both intracellular and extracellular stages to maintain a long-term infection.

### Conclusion

*Salmonella* persistence manifests when a fine balance is achieved between the functions of bacterial virulence factors and host immune clearance mechanisms. One of the more striking observations from the current study is that serovar Typhimurium employs a diverse array of virulence factors to establish and maintain persistence, highlighting the complexity of bacterial persistence. The requirement for numerous virulence factors implies that *Salmonella* utilizes many distinct mechanisms to subvert the immune system. As SPI1 maintains the persistent state of infection, perhaps an equilibrium exists between intracellular and extracellular bacterial forms, and when *Salmonella* SPI1 mutants cannot reestablish an intracellular form, the balance is tipped in the host's favor and *Salmonella* persistence is compromised. This example would represent just one of many balances achieved during *Salmonella* persistence.

The demonstration that certain classes of genes are required at specific times during infection provides a foundation to further dissect *Salmonella* pathogenesis into distinct temporal phases. Because the classes of genes likely reflect a variety of immune system insults that bacteria must counteract, further characterization of the temporal phases of persistence and delineation of the in vivo functions of virulence factors will serve as a powerful approach to understanding the basic principles of *Salmonella* persistence.

## Materials and Methods

### Bacterial strains.

All bacteria were grown and maintained at 37 °C with aeration in Luria-Bertani (LB) broth or on LB agar plates using the following antibiotic concentrations unless otherwise stated: kanamycin (Km) 40 ug/ml, chloramphenicol (Cm) 10 μg/ml, tetracycline (Tc) 10 μg/ml, and streptomycin (Sm) 200 μg/ml. Serovar Typhimurium strains used in this study are derived from wild-type strain SL1344 [69]. The *orgA*::Tet mutant, BJ66, has previously been described [54]. All other deletion mutants (Table 4) were generating using the methods of Datsenko and Wanner [70]. The chloramphenicol acetyltransferase cassette was amplified from pRY104 [71]. Oligos used for the generation of deletion mutants are detailed in Table S2 as well as available at http://falkow.stanford.edu/whatwedo/supplementarydata. Mutations were originally made in the serovar Typhimurium LT2 background, verified by PCR, then transduced into SL1344 using standard P22 transduction methods.

**Table 4:**
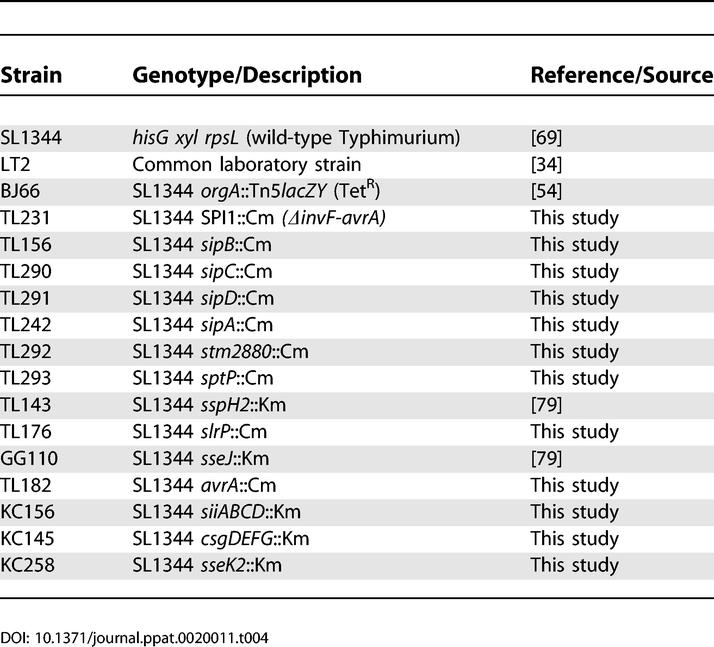
S. enterica Serovar Typhimurium Strains Used in This Study

The serovar Typhimurium SL1344 50,000 CFU transposon-mutagenized library has been described [26]. Briefly, the plasmid pJA1 (a kind gift from Vasudeo Badarinarayana) containing a mini-Tn10 transposase under the control of a isopropyl-B-D-thiogalactopyranoside (IPTG)-inducible promoter was mobilized into SL1344 from E. coli SM10 λ*pir* by a standard mating reaction that was allowed to proceed overnight in the presence of IPTG without antibiotic selection. The next day, the mating reaction was scraped into 10 mM MgSO_4_ and dilutions plated on agar containing 200 μg/ml streptomycin (to select for SL1344) and 30 μg/ml kanamycin (to select for the transposon) to enumerate the number of successful transposition events. Approximately 50,000 colonies were then scraped off of the plates and stored as a single pool in 15% glycerol. Each mutant within the library was produced by the random insertion of the mini-Tn10 cassette, and each copy of the cassette possesses a T7 transcriptional promoter at its end. The T7 promoter allows for transcription into the adjacent genomic region, creating a template to uniquely identify each mutagenized gene [26]. An inoculating loop was used to transfer bacteria from the glycerol stock to LB broth with antibiotics for overnight growth with aeration prior to inoculating into mice.

### Passage of serovar Typhimurium transposon library through 129X1/SvJ mice.

The transposon-mutagenized library was passaged two independent times through mice. In the first passage, 50 female 129X1/SvJ mice (The Jackson Laboratory, Bar Harbor, Maine, United States), 7–9 wk old, were each injected IP with 1.6 × 10^4^ CFU from an overnight culture of the library. Ten mice were sacrificed at 7, 14, and 21 d postinfection, eight mice at 28 d, and four mice at 49 d. Spleens and livers were removed and homogenized, and then the entire homogenate for each organ was plated on LB agar containing streptomycin and kanamycin in 150-mm diameter petri dishes. Dilutions were also plated in order to enumerate bacterial loads. Colonies were scraped off of the plates into PBS, the bacteria pelleted, and the pellets resuspended in 5-ml 20% glycerol. The initial input inoculum was also plated and collected to account for the effect this step may have on the complexity of the library [27]. For each time point, a pool of all the samples of a given tissue was generated by combining 1 ml from each of the resuspended bacterial slurries. One-ml aliquots were frozen at −80 °C. Thus, for each time point, there are bacterial stocks from each tissue of each individual mouse, as well as a pool of either the spleen or liver samples.

In the second passage, ten mice were inoculated with 7.9 × 10^3^ CFU from an overnight culture of the library. This was approximately 2-fold fewer bacteria than were inoculated in the first passage. Seven mice survived the acute phase of the disease and were sacrificed at 29 d postinfection. Bacteria were recovered and enumerated from their spleens and livers, a pool was generated for each organ, and the inoculum was replated as described above.

### Microarray hybridizations.

Phenol/chloroform genomic DNA (gDNA) extractions, HinP1I digestions, T7 transcriptions, and preparation of samples for microarray hybridization were performed as described previously [26]. Briefly, 10 μg of gDNA was digested with HinP1I (New England Biolabs, Beverly, Massachusetts, United States) and 2 μg of purified, digested DNA was used as the template for in vitro transcription with the MEGAscript T7 transcription kit (Ambion, Austin, Texas, United States) according to the manufacturer's protocol except that all reaction volumes and reagents were doubled. After overnight transcription, the sample was DNase treated, the RNA recovered, and 1.5–2 μg subject to a reverse transcription reaction with random hexamers as primers using Superscript III(-) (Invitrogen Life Technologies, Carlsbad, California, United States). Aminoallyl-dUTP (Sigma-Aldrich) incorporation was performed in a separate Klenow reaction (New England Biolabs) followed by CyDye (Amersham Biosciences, Little Chalfont, United Kingdom) conjugation as described previously [72]. All samples were hybridized against a common reference in which gDNA from a separate overnight culture of the library was used as the template for the in vitro transcription reactions. The reference was conjugated to Cy3 whereas all the samples were conjugated to Cy5.

For the first passage, hybridizations were performed representing input and pooled 7, 14, 21, 28, and 49 d postinoculation spleen samples. For the second passage, hybridizations were performed for the liver and spleen samples of each individual mouse, pooled samples for the two tissues, as well as the input sample. All hybridizations were performed using a second generation microarray that had been constructed as described previously [73], except that LT2 gDNA was used as the PCR template for generating the serovar Typhimurium products (5,727 probes) that were spotted on the array. In addition S. enterica serovar Typhi–specific probes from three unique strains were added to generate a non-redundant microarray for both serovars: CT18 gDNA was used to generate probes specific to serovar Typhi strain CT18 (433 additional probes) and the serovar Typhi–specific plasmids pHCM1 (55 probes) and pHCM2 (3 probes) [74]; Ty2 gDNA was used to generate probes for serovar Typhi strain Ty2 specific genes (13 probes) [75]; and 403Ty (a generous gift from Dr. Bruce Stocker, Stanford University) gDNA was used for the z66 flagellar antigen probes (21 probes). PCR primers for these new probes were selected using Microarray Architect [76] that is designed to identify regions of each open reading frame (ORF) without cross-homology to other ORFs present on the microarray. The primers were selected to match melting temperatures and optimal PCR product lengths of 150–300 base pairs. Hybridizations were performed on this second generation array as described previously except that they were allowed to proceed at 55 °C [73]. Two microarrays were hybridized for each of the pooled spleen samples except for the input and 28 d samples that were represented by three microarrays each.

### Data analysis.

Data were downloaded from the Stanford Microarray Database (SMD; http://genome-www5.stanford.edu) [77]. The Typhi-specific features on the second-generation microarray served as a means of assessing the specificity of hybridization. An appropriate Cy3 net mean intensity filter was therefore applied to each downloaded dataset to ensure that features (spots) with low signal intensities in this channel were not included in the analysis. When the appropriate Cy3 filter was applied, very few Typhi-specific features made it through to the analysis. An additional regression correlation greater than 0.6 filter was also applied as a measure of feature quality and, depending upon the dataset, features that were missing in 20%–40% or more of the arrays were removed. Further details regarding the filters applied to each dataset are indicated in the text. The microarray datasets have been made available for download from SMD.

A SAM (Statistical Analysis of Microarrays, version 1.21; [33]) two-class analysis was implemented in order to identify the genes that were most significantly different between the input and samples. Negative significant genes are genes whose mutants are absent in the tissue samples but present in the input. The two-class analysis that was performed on the data from the first passage included five arrays corresponding to the pooled spleen sample and three input arrays. For the second, 29-d passage, individual spleen and liver samples as well as pooled and input samples were analyzed by SAM (see text for further details). The overlap between the first and second passage was determined. Because there are multiple spots corresponding to many of the Typhimurium genes, overlap between the two biological replicates was assessed on a spot-by-spot basis rather than by genes. The false discovery rate (FDR) is calculated within the SAM analysis program and determines the proportion of each SGL that is identified by chance and therefore falsely discovered. A 1% FDR cutoff essentially generates an SGL in which approximately 1% of the genes will be falsely called (either false negatives or false positives). Application of the 1% FDR generated SGLs for both microarray datasets (both passage 1 and passage 2) that have approximately 300 negative significant genes/features in addition to a few positive significant ones. This is why the top 300 negative significant features in either dataset were analyzed for overlap. There were no overlapping positively significant genes, indicating that no mutants consistently had a growth advantage between the two passages

When applicable, LACK analysis was employed to assess the likelihood that any enrichment in a particular class of genes was due to chance [78]. Additional data manipulation and analyses were performed using Microsoft Excel, Microsoft Access, (Microsoft, Redmond, Washington, United States) and analysis tools available at http://falkow.stanford.edu/whatwedo/software.

In order to identify genes that were significantly different as a function of time, pair-wise groupings of arrays from a dataset that included the input arrays as well as the spleen pool arrays from all time points were analyzed using a number of SAM two-class analyses for every pair-wise combination. Significant genes were then assembled into a single file for further analysis. This dataset, along with the 28-d SAM analysis, has been made available as supplementary tables available for download from http://falkow.stanford.edu/whatwedo/supplementarydata as noted in the text.

### CI mouse infections.

For each mutant and time point of interest, five female 129X1/SvJ mice were inoculated IP with 1 × 10^4^ CFUs in 200 μl PBS containing an equal proportion of wild-type SL1344 and one of the mutants, all of which were individually grown overnight with aeration. Dilutions of the inoculum were plated on both streptomycin plates (which selects for SL1344) and streptomycin plates supplemented with kanamycin, chloramphenicol, or tetracycline (to select for the deletion mutant) in order to verify the dose and ratio of wild-type to mutant bacteria. At the appropriate timepoints, four to five mice were sacrificed and tissues aseptically removed and homogenized. Homogenates from MLN, liver, and spleen were plated on streptomycin plates or plates containing streptomycin supplemented with chloramphenicol, tetracycline, or kanamycin. Enumeration of wild-type bacteria and mutant bacteria allowed for the determination of the CI ratio between wild-type and mutant bacteria using the following formula: CI = (mutant output/wild-type output)/(mutant input/wild-type input). Standard errors were calculated and significance of results determined by application of the Mann-Whitney statistical test on the log10 value of the CI ratios.

## Supporting Information

Dataset S1 Dataset for the Analysis of Time-Dependent Negative Selection of Serovar Typhimurium Mutants (58 KB TXT)

Figure S1 Plot Generated from the SAM Analysis Comparing Passage 2 Liver and Spleen Samples A SAM two-class analysis was performed comparing the liver and spleen samples to one another, including pooled and individual mouse samples, in order to determine if there were any features that were statistically different between the two. The linearity of the plot indicates that there were few, if any, features whose data indicated a significant difference between the two organs suggesting that there is no major bottleneck during the seeding of either organ or that there are gene differentially required by serovar Typhimurium to survive in either systemic site.
(329 KB PDF)

Table S1 Complete List of Negatively Selected Serovar Typhimurium Genes (397 KB XLS)

Table S2 Oligos Used in This Study to Create Serovar Typhimurium Mutants (76 KB DOC)

## Abbreviations

CFU: colony-forming unit
CI: competitive index
COG: Clusters of Orthologous Groups of proteins
IP: intraperitoneal
MLN: mesenteric lymph nodes
OM: outer membrane
ORF: open reading frame
PMN: polymorphonuclear lymphocytes
SAM: Statistical Analysis of Microarrays software
SGL: significant gene list
SPI: *Salmonella* pathogenicity island
T3SS: type III secretion system
