# Supplementary figures and images for: Genome-Wide Screen for Salmonella Genes Required for Long-Term Systemic Infection of the Mouse
Source: PLoS Pathog. 2006 Feb 24;2(2):e11. doi: 10.1371/journal.ppat.0020011 (PMC1383486; doi:10.1371/journal.ppat.0020011)

SAM plot for liver versus spleen comparison

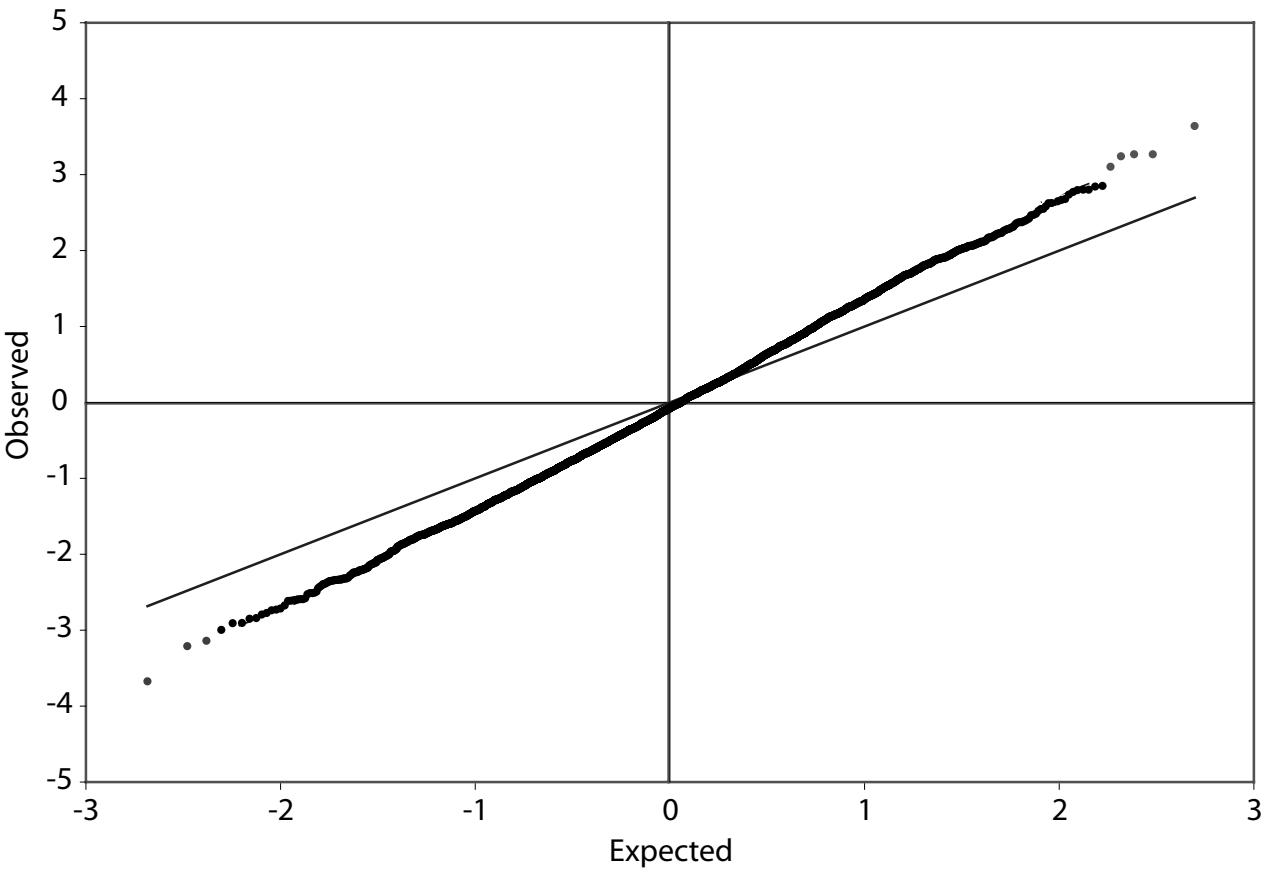

Supplement: Figure S1 — A SAM two-class analysis was performed comparing the liver and spleen samples to one another, including pooled and individual mouse samples, in order to determine if there were any features that were statistically different between the two. The linearity of the plot indicates that there were few, if any, features whose data indicated a significant difference between the two organs suggesting that there is no major bottleneck during the seeding of either organ or that there are gene differentially required by serovar Typhimurium to survive in either systemic site. (329 KB PDF) [file ppat.0020011.sg001.pdf]
