# Supplementary material for: Genome-Wide Screen for Salmonella Genes Required for Long-Term Systemic Infection of the Mouse
Source: PLoS Pathog. 2006 Feb 24;2(2):e11. doi: 10.1371/journal.ppat.0020011 (PMC1383486; doi:10.1371/journal.ppat.0020011)
Supplement: Table S2 — (76 KB DOC) [file ppat.0020011.st002.doc]

Table S2. Primers used for the generation of deletion mutants.

| STMID/  Mutant Name^a^ | Primer name | Primer sequence |
| --- | --- | --- |
| STM 0800/  slrP::Cm | slrP::Cm for | Gcctcaacagaggtgcctttaaaagaagagatatggaataaaataagtgcgcggtgttcctttccaag |
|  | slrP::Cm rev | ctcttttttcagcaatatattctccatgatctcagtaaagagggtctgctgggattttatttattcagc |
|  | slrP for | gaaataatgacgactgtgacc |
|  | slrP rev | tgcgctggaaaaagcgctac |
| STM 1139-1142/  csgDEFG::Km | csgDEFG::Kan for | CAGCTGTCAGATGTGCGATTAAAAAAAGTGGAGTTTCATCGTGTAGGCTGGAGCTGCTTC |
|  | csgDEFG::Kan rev | AAAAAAGCGTGGGGTTCTTCCCCACGCTTTGTCGTATTCAATTCCGGGGATCCGTCGACC |
|  | csgDEFG for | GTGCCTTTATTTTATGGGGG |
|  | csgDEFG rev | GAATACGGCGGTCACAAAAT |
| STM 1631/  sseJ::Km | sseJ::Kan for | TTATTTGCTAAAGCGTGTTTAATAAAGTAAGGAGGACACTGTGTAGGCTGGAGCTGCTTC |
|  | sseJ::Kan rev | AGCTGTGTTTTGCTCAAGGCGTACCGCAGCCGATGGAACTATTCCGGGGATCCGTCGACC |
|  | sseJ for | TGCTAATATTTGCTAATTAA |
|  | sseJ rev | CCACCGGCACTATGATATTG |
| STM 2137/  sseK2::Km | sseK2::Kan for | AAATTAAGGTTAAAAACTGATAATTTAAGCGTGTAAAAATGTGTAGGCTGGAGCTGCTTC |
|  | sseK2::Kan rev | CGTGACGGTGGGAAGGGTGAGTAAAACAAGGCTATCATGAATTCCGGGGATCCGTCGACC |
|  | sseK2 for | CAAAAAGTTTCCAGGTTATT |
|  | sseK2 rev | AACTCTGGATACCGATTATA |
| STM 2241/  sspH2::Km | sspH2::Kan for | CGGACAGATACTATATGTAAATTTATAAAGGTTTTTTGTTGTGTAGGCTGGAGCTGCTTC |
|  | sspH2::Kan rev | GGAATATCTTTGTCGCACCGCACCTCATTCACCTGGTGCAATTCCGGGGATCCGTCGACC |
|  | sspH2 for | CAGGAAGAGATGATGTCTGC |
|  | sspH2 rev | TGTCTGATGTTGTTCGTCCG |
| STM2865-2900/  spi1::Cm | SPI1::Cm for | aatatggtcttaattatatcatgatgagttcagccaacggtgatatggccgcggtgttcctttccaag |
|  | SPI1::Cm rev | gtcttatggcgctggaaggatttcctctggcaggcaaccttataatttcactgggattttatttattcagc |
|  | SPI1 for | aacgtaagagacaaatggcc |
|  | SPI1 rev | gctattcaggaaacatacg |
| STM 2865/  avrA::Cm | avrA::Cm for | ATATTTTTGCAGGCAATATATTAAATCTGAAAAGTTAAAGGTGTAGGCTGGAGCTGCTTC |
|  | avrA::Cm rev | GCTGGAAGGATTTCCTCTGGCAGGCAACCTTATAATTTCAATTCCGGGGATCCGTCGACC |
|  | avrA for | CCTGGCTCAATCATTGAGGC |
|  | avrA rev | ATATAGAGTAGTCTTATGGC |
| STM 2878/  sptP::Cm | sptP::Cm for | caaaaacatactgcaggaatatgctaaagtatgaggagagaaaatgcggtgttcctttccaag |
|  | sptP::Cm rev | gcattctattgttccgtgaatcccggaaatctgcacgtacctgctcctgggattttatttattcagc |
|  | sptP for | atatgcatgcggagtgaaacgatgctaaagtatgaggagag |
|  | sptP rev | atatgtcgactcagcttgccgtcgtcataag |
| STM 2880/  stm2880::Cm | stm2880::Cm for | ttctctcgcttgacaacaattgaataagattaattgttatgaaaatcgcggtgttcctttccaag |
|  | stm2880::Cm rev | gtgtgcttgcaatttactttcctcttgaattatatcttttataagactgggattttatttattcagc |
|  | stm2880 for | sipA for primer used |
|  | stm2880 rev | sptP rev primer used |
| STM 2882/  sipA::Cm | sipA::Cm for | agatcaaaacgcaggccacgaatcttgcggcgaatctttccgcagtcagagcggtgttcctttccaag |
|  | sipA::Cm rev | ccgataaggccgtccagcacattacttaacacatagtcagtttccccttctgggattttatttattcagc |
|  | sipA for | agctgcctgaaatcttatgg |
|  | sipA rev | atcaacatcaacggcaatac |
| STM 2883/  sipD::Cm | sipD::Cm for | tcatccggggatcgttgccgaacggccgcagactccctcggcgagcggcggtgttcctttccaag |
|  | sipD::Cm rev | cggtttccaggctgctacttatcgtactgctcagcacttttaccaggctgggattttatttattcagc |
|  | sipD for | atatgcatgcggagtgaaacgcgaactggggatattatgc |
|  | sipD rev | atatgtcgactgttatccttgcaggaagc |
| STM 2884/  sipC::Cm | sipC::Cm for | cttatttaaataatcattctgttgagaatagttcacagacagcttcggcggtgttcctttccaag |
|  | sipC::Cm rev | cagattaagcgcgaatattgcctgcgatagcagcgagtgcggatgctctgggattttatttattcagc |
|  | sipC for | atatgcatgcggagtgaaacgatgttaattagtaatgtggg |
|  | sipC rev | atatgtcgacttaagcgcgaatattgcc |
| STM 2885/  sipB::Cm | sipB::Cm for | gcggcggataaagcttttaaagatgtggtggcaacgaaagcgggcgacctgcggtgttcctttccaag |
|  | sipB::Cm rev | ttgtttaagccactgctgaatctgatccatggcaaaacgggcgagcatactgggattttatttattcagc |
|  | sipB for | gtgacctttatgcagtagcg |
|  | sipB rev | aacggcactggaagacattc |
| STM 4257-4260/  stm4257-4260::Km | stm4257-4260::Kan for | ACAAAAACATTTTATTCACAATGTAATATCAGGAGACAACGTGTAGGCTGGAGCTGCTTC |
|  | stm4257-4260::Kan rev | AATTTTGATCGGCAAAAAACTTTTGTATGCTTTTATTTCCCATATGAATATCCTCCTTAG |
|  | 4257-4260 for | TCCTTTATCCTTTCTAATCC |
|  | 4257-4260 rev | AGAAGATAAATCAATTACAG |

^a^ The serovar Typhimurium mutants SPI1::Cm, sipB::Cm, sipC::Cm, sipD::Cm, sipA::Cm, stm2880::Cm, sptP::Cm, slrP::Cm were constructed by adding a chloramphenicol acetyltransferase cassette (Cm) (M35190 accession number) described in Wang and Taylor 1990. Gene 94 (1), 23-28 (1990). The remaining mutants were constructed by inserting a kanamycin resistance cassette from vector pKD3 described in Wanner and Datsenko. Underlined bases correspond to the portion of each oligonucleotide that hybridizes to the PCR template.
